# Optimisation of Reference Genes for Gene-Expression Analysis in a Rabbit Model of Left Ventricular Diastolic Dysfunction

**DOI:** 10.1371/journal.pone.0089331

**Published:** 2014-02-18

**Authors:** Walid Nachar, David Busseuil, Yanfen Shi, Teodora Mihalache-Avram, Mélanie Mecteau, Eric Rhéaume, Jean-Claude Tardif

**Affiliations:** 1 Montreal Heart Institute and Université de Montréal, Montreal, Quebec, Canada; 2 Department of Medicine, Université de Montréal, Montreal, Quebec, Canada; Scuola Superiore Sant'Anna, Italy

## Abstract

Left ventricular diastolic dysfunction (LVDD) is characterized by the disturbance of ventricle’s performance due to its abnormal relaxation or to its increased stiffness during the diastolic phase. The molecular mechanisms underlying LVDD remain unknown. We aimed to identify normalization genes for accurate gene-expression analysis of LVDD using quantitative real-time PCR (RT-PCR) in a new rabbit model of LVDD. Eighteen rabbits were fed with a normal diet (n = 7) or a 0.5% cholesterol-enriched diet supplemented with vitamin D2 (n = 11) for an average of 14.5 weeks. We validated the presence of LVDD in this model using echocardiography for diastolic function assessment. RT-PCR was performed using cDNA derived from left ventricle samples to measure the stability of 10 genes as candidate reference genes (*Gapdh, Hprt1, Ppia, Sdha, Rpl5, Actb, Eef1e1, Ywhaz, Pgk1, and G6pd)*. Using geNorm analysis, we report that *Sdha*, *Gapdh* and *Hprt1* genes had the highest stability (M <0.2). By contrast, *Hprt1* and *Rpl5* genes were found to represent the best combination for normalization when using the Normfinder algorithm (stability value of 0.042). Comparison of both normalization strategies highlighted an increase of natriuretic peptides (*Bnp* and *Anp*), monocytes chemotactic protein-1 (*Mcp-1*) and NADPH oxidase subunit (*Nox-2*) mRNA expressions in ventricle samples of the hypercholesterolemic rabbits compared to controls (*P*<0.05). This increase correlates with LVDD echocardiographic parameters and most importantly it molecularly validates the presence of the disease in our model. This is the first study emphasizing the selection of stable reference genes for RT-PCR normalization in a rabbit model of LVDD.

## Introduction

Left ventricular diastolic dysfunction (LVDD) accounts for almost half of the cases of heart failure in clinical practice [1]. It is characterized by abnormal left ventricle (LV) relaxation or reduced ventricular compliance, which leads to increased filling pressures [2]. Treatments for LVDD are currently limited to diuretics for symptom relief and to those addressing the underlying cause, including arterial hypertension, myocardial ischemia and aortic valve stenosis (AVS). Many studies have demonstrated the association of hypercholesterolemia with atherosclerosis and plaque formation [3]. However, few studies reported the impairment of cardiac function in humans and animals after exposure to a hypercholesterolemic diet [4]–[7]. To understand the genetic and molecular mechanisms underlying the cardiac impairments observed in LVDD, numerous studies assessed the expression of genes that might be related to this pathology [8]–[10]. The level of brain natriuretic peptide (BNP), a neurohormone secreted by the ventricle in response to ventricular volume expansion and increased LV filling pressure, has been shown to be increased in patients with LVDD [11] and animal models with accompanying LV hypertrophy [9], [10]. Serum BNP levels have indeed been proposed as a biomarker of LVDD severity [12] and a link between LVDD and LV *Bnp* mRNA expression has been established [9], [13]. Oxidative stress and inflammation processes have also been reported to contribute to DD development [14], [15]. Indeed, numerous studies showed an increase of the expression of inflammatory genes such as *Mcp-1* or *IL-6* and contributors to oxidative stress such as NADPH oxidase and its subunits in DD cases [9], [10]. Although large animal models have been described for LVDD [16], only a limited number of models of experimental LVDD have been described in rabbits. Among these models, the development of LVDD with age or hypercholesterolemia in rabbits has been shown to be similar to the changes seen in humans [4], [5], [17], making rabbits a valid model to study the mechanisms underlying this pathology.

mRNA quantification by real-time PCR (RT-PCR) remains the most common method used to study gene expression changes associated with different pathologies. RT-PCR is a very sensitive method; however, accurate gene normalization is essential for data interpretation [18], [19]. Multiple strategies are available for normalization and can affect the quality of gene-expression studies [19]–[21]. However, differences between animal models, tissues, cell types, experimental conditions and protocols require the specific evaluation of different reference genes for the selection of the best ones in each experimental setup. Experimental data have clearly demonstrated the value of using multiple reference genes to normalize expression data [21]. The aim of the current study was to identify and validate suitable stable reference genes that can be used for gene-expression studies in a new LVDD rabbit model. These genes may be ultimately used to study gene expression and mechanisms in relation to different possible therapeutic approaches using this model.

## Methods

### Animals

Animal care and procedures complied with the Canadian Council on Animal Care guidelines and were approved by the Montreal Heart Institute Research Center ethics committee for animal research. To study the effect of the diet on the progression of LVDD, 18 New-Zealand White rabbits (2.91±0.04 kg) were fed with a normal diet (n = 7) or a 0.5% cholesterol-enriched diet supplemented with vitamin D_2_ (50 000 IU.day^−1^) in the drinking water (n = 11) for an average period of 14.5 weeks. Cholesterol-diet rabbits were then fed with a standard diet (without cholesterol and vitamin D_2_) to mimic cholesterol-lowering therapy for 2 weeks. Animals were then killed and the LV were removed, flushed with sterile saline, snap frozen in liquid nitrogen and stored at –80°C.

### Echocardiography

Examinations were carried out with a phased-array probe 10S (4.5 ∼ 11.5 Mega Hertz) using a Vivid 7 Dimension system (GE Healthcare Ultrasound, Horten, Norway). Intra-muscular injections of ketamine (90 mg/kg) and midazolam (0.75 mg/kg) were used for sedation. Complete echocardiography-Doppler examinations were performed at baseline, on the day of the switch to normal diet and on the day of sacrifice to assess several LVDD parameters. Pulsed-wave Doppler was used to record transmitral flow (TMF), left and right pulmonary venous flow in an apical 4-chamber view. Peak velocity during early filling (E), mitral E-wave deceleration time, deceleration rate, peak velocity during active atrial filling (A), and mitral A-wave duration were measured, and E/A ratio was calculated on TMF. Peak velocity of systolic flow (S), diastolic flow (D), reversed atrial flow (Ar), and Ar wave duration were measured, and S/D ratio was calculated on both left and right pulmonary venous flow. Mitral A wave duration minus left (and right) pulmonary venous flow Ar wave duration (A-Ar_left_ and A-Ar_right_ duration) were also calculated from TMF and pulmonary venous flow. Mitral annulus velocities during early filling (Em) and active atrial filling (Am) in both lateral and septal annulus were measured by tissue Doppler imaging. Em/Am and E/Em ratio were calculated. LVDD was classified using previously published criteria [5], [22], [23]. Special care was taken to obtain similar imaging planes on serial examinations by reviewing previous recordings before the follow-up study. All echocardiographic imaging and measurements were performed throughout the protocol by the same experienced investigator blinded to diet allocation. The average of 3 consecutive cardiac cycles was used for each measurement.

### RNA Extraction and cDNA Synthesis

LV tissue (15 to 30 mg) was homogenized in lysis buffer using “PowerGen 125” homogenizer (Fisher Scientific, Ontario, Canada) for 30 seconds. Total RNA was extracted using Qiagen RNeasy Fibrous Tissue Mini Kit (Qiagen, Ontario, Canada), according to manufacturer’s instructions. Genomic DNA contamination was removed by RNase-free DNase I digestion during the extraction procedure (according to the RNA extraction kit protocol). The quality and quantity of total RNA was assessed using Agilent RNA 6000 Nano Kit for Bioanalyzer 2100 System (Agilent Technologies, Santa Clara, California, USA). RNA integrity numbers (RIN) were higher than 8.8 for all samples. 700 ng of total RNA were used for first strand synthesis of complementary DNA (cDNA) using High Capacity cDNA Reverse Transcription Kit with RNase inhibitor (Applied Biosystems, California, USA) in a final reaction volume of 20 µl according to manufacturer’s instructions.

### mRNA Quantification Using Real-time PCR

Quantitative RT-PCR experiments were performed using Stratagene MX 3005P thermal cycler (Agilent Technologies, Santa Clara, California, USA). Final reaction volume was 25 µl and composed of 1X SyberGreen (GoTaq® qPCR Master Mix, Promega Corporation, Madison, WI, USA or Perfecta SyberGreen FastMix®, ROX ^TM^, Quanta BioSciences, Gaithersburg, USA), reference ROX dye, 1 µM of forward and reverse primer, and 2.16 ng of cDNA template. A first incubation was made at 50°C for 2 min followed by an initial denaturation at 95°C for another 2 min. Amplification was then performed during 40 cycles of denaturation at 95 °C for 15 sec and annealing/extension at 60 °C for 1 min. At the end, a dissociation curve was produced to analyze and confirm the specificity of the amplification by observation of a single peak in the curve. Standard curves of five points were produced for each gene to transform sample’s Cts to relative expression values. Amplification efficiencies were calculated using MxPro software (version 4.10) to analyze the slopes of the standard curves according to Pfaffl [24] and are presented in Tables 1a and 1b. All samples were run in duplicate and the mean values were used for calculations. PCR products were initially sequenced to confirm the specificity of amplifications. Relative quantities of the unknown samples were normalized against the normalization factor (NF) which was calculated from the geometric means of the expression of the best three and two reference genes selected by geNorm or Normfinder algorithm respectively.

**Table 1:** Primer sequences and their characteristics for the 10 reference genes whose variation in expression was assessed using RT-PCR.

Gene Symbol	Accession number	Forward primer ( 5'-3' )	Reverse primer ( 5'-3' )	PCR efficiency (%)	Amplicon length (bp)	Tm (°C)
***Gapdh***	NM_001082253	TGGTGAAGGTCGGAGTGAAC	ATGTAGTGGAGGTCAATGAATGG	94.2	121	75.7
***Hprt1***	NM_001105671	CCTTGGTCAAGCAGTATAATC	GGGCATATCCTACAACAAAC	96.1	135	74.0
***Ppia***	NM_001082057	ACTTCACACGCCACAATG	TGATCTTCTTGCTGGTCTTG	96.6	273	79.2
***Sdha***	XM_002723194	GGACCAGGACGCCATCCACTAC	TCCACCGAACGCACGCTGATAG	96.1	124	76.6
***Eef1e1***	XM_002714163	ACCGCAGAAGAGAAAGCCATAG[23]	AGCGATGTAGCCCATAGTAGAGGA	91.8	190	76.4
***G6pd***	NM_001171382	GCAGAGTGAGCCCTTCTTC	GCCAGCCACATAGGAGTTG	93.1	85	75.6
***Pgk1***	XM_002720132	TGTTGGTCGGGCGAAGCAG[23]	CAGTGTCTCCACCGCCGATG	92.2	149	77.9
***Rpl5***	NM_001195679	GATTGCGTATGCCCGTATAG	CTCCAGTCACCTCCACTTG	98.0	194	76.7
***Actb***	NM_001101683	CAAGCGTGGCATCCTGAC	CTCGTTGTAGAAGGTGTGGTG	98.0	100	76.5
***Ywhaz***	XM_002710718	GGTCTGGCCCTTAACTTCTCTGTGTTCTA [25]	GCGTGCTGTCTTTGTATGATTCTTCACTT	103.8	142	73.1

Amplification efficiencies were calculated using MxPro software to analyze the slope of the standard curve according to Pfaffl [24].

GeNorm analysis determines the pairwise variation of a gene with the other tested genes, through calculation of standard deviation of the log-transformed expression ratios, and defines the internal control gene-stability measure M as the average pairwise variation of a particular gene with all other genes [21]. The gene with the most stable expression gene has the lowest M value. A stepwise exclusion of the least stable gene allows a recalculation of the M values for the rest of genes. To determine the optimal reference gene number for normalization, we considered 0.15 as the cut-off for pairwise variation (V_n_/_n+1_), between NF_n_ and NF_n+1_ as recommended by Vandesompele et al [21].

Normfinder is a mathematical model of gene expression that enables estimation of the overall variation of the candidate normalization genes and of the variation between sample subgroups of the sample set [20]. Indeed, Normfinder selects the best gene for normalization based on its intra- and inter-group variation and combines the two into a stability value. It also determines the best combination of two genes which are chosen with a similar-sized fold change but in the opposite direction.

### Reference Gene Selection

Ten candidate genes were tested based on their common use in literature as reference genes [20], [21], [25]. Gene-specific, intron-spanning whenever possible, primer pairs were designed using Beacon Designer software, version 7.5 (Premier Biosoft International) or adapted from earlier publications [25]. Primer characteristics are described in Tables 1 and 2 for reference genes and genes of interest, respectively. A minimum of two designed primer pairs were tested for optimization. Primers were synthesized by Integrated DNA Technologies Inc (California, USA). Gene expression stability was tested using the geNorm algorithm which is included in the qbase Plus 2 software (version 2.3, Biogazelle, Zwijnaarde, Belgium), and Normfinder which is a free Visual Basic application (VBA) for Excel.

**Table 2:** Primer sequences and their characteristics for the genes of interest whose variation in expression was assessed using RT-PCR.

Gene Symbol	Accession number	Forward primer ( 5'-3' )	Reverse primer ( 5'-3' )	PCR efficiency (%)	Amplicon length (bp)	Tm (°C)
*Anp*	NM_001082262	GTACAACGCCATGTCCAAC	CTTCATCACTCTGCTCACTTAG	95.9	125	74.5
*Bnp*	XM_002724454	CCTGTGCCCCTGGATGAG	CCGAAGCAGCCTGAGTCC	100.5	80	80.2
*Mcp-1*	NM_001082294	CAGTGAAGAGGCTAATGAG	GGTTGTGGAATAAGAGGTC	100.2	188	76.2
*Nox-2*	AF323788	AATGCTTGTGGCTGTGATAAG	TACCAGACTGACTTGAGGATG	98	176	75.9

Amplification efficiencies were calculated using MxPro software to analyze the slope of the standard curve according to Pfaffl [24].

### Statistics

Analyses were performed with SAS version 9.3 (SAS Institute Inc., Cary, NC, USA) and Prism graph pad version 5.04. All analyses were conducted at the 0.05 significance level. Data are presented as the mean ± standard error of the mean (SEM). For echocardiographic parameters, analysis of covariance (ANCOVA) adjusted for the baseline value was used to assess the change over the 3 time points in each treatment group and to compare groups at each time points separately. The non-parametric Mann-Whitney test was used to compare mRNA levels of groups. Spearman correlation was used to assess correlations between mRNA and echocardiographic data.

## Results

### LVDD Characterization Using Echocardiography in a Hypercholesterolemic Rabbit Model

LVDD has previously been observed in hypercholesterolemic rabbits [4]. Given our prior experience with a hypercholesterolemic rabbit model that develops aortic valve stenosis [26], and since LVDD is often associated with aortic valve stenosis, we performed a detailed characterization of diastolic heart function in this animal model using echocardiography. We observed an increase in E wave velocity, E wave deceleration rate, mitral annulus lateral E/Em ratio, septal annulus E/Em ratio and left and right pulmonary vein Ar durations after 14.5 weeks of hypercholesterolemic diet and two weeks after ending this diet compared with the normal diet control group (Table 3). We also report a significant decrease in E wave deceleration time and decreased values of left and right pulmonary vein A-Ar durations in the hypercholesterolemic group at these two time-points compared with the normal group (Figure S1 in File S1). LVDD was maintained even after the change for two weeks from a cholesterol-enriched diet to a normal diet, as observed by the negligible changes of diastolic dysfunction-related parameters reported in Table 3.

**Table 3:** Statistically significant altered left ventricular diastolic function echocardiographic parameters in hypercholesterolemic compared to normal rabbit groups.

	Baseline		14.5 wks		14.5+2 wks	
	Normal group	Hyperchol group	Normal group	Hyperchol group	Normal group	Hyperchol group
E wave velocity (cm/s)	66.2±4.4	62.6±4.2	67.3±6.3	117.7±6.4***	72.9±2.5	100.9±8.5*
E-wave deceleration time (ms)	35.1 ±1.7	33.9±2.4	32.8±3.3	24.0±2.1*	39.0± 3.4	23.2±2.0***
E-wave deceleration rate (m/s^2^)	19.2±1.6	20.4±2.2	20.9±1.3	51.2±3.5***	19.5 + 1.8	46.2±5.2***
Lateral annulus E/Em ratio	6.4±0.6	7.0±0.8	6.8±0.8	12.0 ±1.3*	6.2±0.6	12.7±1.5**
Septal annulus E/Em ratio	6.2±0.5	8.1±0.8	7.4±0.7	10.7±0.8*	8.2±0.5	12.1±0.7*
Left pulmonary vein A- A_r_ durations (ms)	–4.8±3.3	–2.3±3.6	–7.1±1.5	–26.5±6.5*	–5.1±1.5	–27.6±4.5**
Right pulmonary vein A- A_r_ durations (ms)	–2.9±3.5	–0.2±3.5	–7.0±2.3	–24.0±3.0**	–3.4±1.5	–23.4±4.9***
Left pulmonary vein A_r_ duration (ms)	43.2±2.5	39.2±2.7	40.5±1.2	51.4±4.5*	39.7±1.5	53.3±2.7***
Right pulmonary vein A_r_ duration (ms)	41.2±2.1	35.8±1.6	40.4±2.2	50.1±2.1**	38.1±1.4	51.3±3.3***

Comparison between the normal and hyperchol group at the given time-point. After baseline echocardiographic assessment, the hyperchol group was exposed to a cholesterol-enriched diet + vitamin D for 14.5 weeks before being switched for 2 weeks to a normal diet. E: peak velocity during early filling; Em: mitral annulus velocities during early filling in both lateral and septal annulus; A: peak velocity during active atrial filling; Ar: reversed atrial flow; A­Ar durations: mitral A-wave duration minus left (or right) pulmonary venous flow Ar wave duration. N = 7 for the normal group and n =  7 to 11 for the hyperchol group. * *P*<0.05, **P<0.01, ***P<0.001 for comparison between the normal and hyperchol group at the given time-point.

### Real-time PCR and Selection of Reference Genes

To assess mRNA expression from ventricle samples obtained from normal and LVDD rabbits, we used GeNorm and Normfinder algorithms to test the stability of 10 candidate reference genes based on their common use in literature [20], [21] (Table 1).

**Figure 1:**
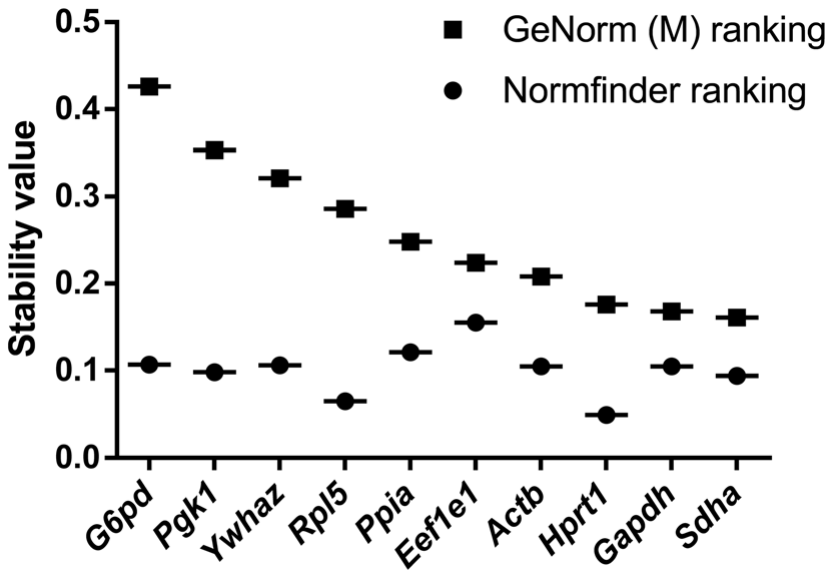
GeNorm and Normfinder ranking of the 10 reference genes tested for the mRNA expression stability in left ventricles of 18 rabbits (normal diet n = 7 and cholesterol diet n = 11). *Sdha*, *Gapdh* and *Hprt1* are shown as the best 3 genes with the lowest geNorm M value (M =  0.161, M =  0.168 and M = 0.176, respectively). Lower M value corresponds to higher gene stability. However, the use of the Normfinder algorithm results in the selection of *Hprt1* (0.049) as the most stable gene and suggests to use the average of *Hprt1* and *Rpl5* (0.042) as normalization factor.

**Figure 2:**
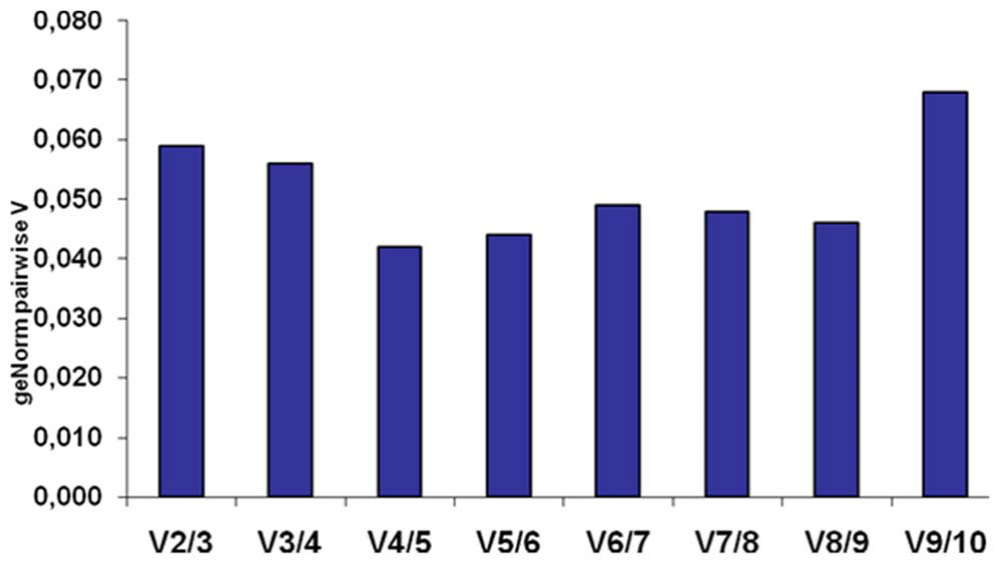
Determination using the geNorm algorithm of the optimal number of reference genes required for effective normalization. Pairwise variation (V_n_/_n+1_) calculated between the normalization factors NF_n_ and NF_n+1_ to assess the optimal number of reference genes to use in normalisation. V<0.15 is considered sufficient to normalise. Here, the use of two to three genes have a V_2_/_3_ = 0.0575, which indicates that the use of two to three genes is sufficient to calculate the normalization factor.

GeNorm analyses are based on pairwise correlation of the expression level for each gene with the rest of the tested genes. An average of expression stability, M value, is given for each gene. The expression of a gene with an M value < 0.5 is considered highly stable. We found that all of the 10 genes tested in the LVs are stably expressed as documented by their average expression stability below 0.5. Among them, the three genes with the lowest M values were *Hprt1* (M = 0.176), *Gapdh* (M = 0.168) and *Sdha* (M = 0.161); see Figure 1 for gene ranking. Pairwise variation (V) analysis, which provides the optimal number of reference genes that should be used in normalization, was also calculated between NF_n_ and NF_n+1_. The geometric mean of the three best genes (*Sdha*, *Gapdh and Hprt1*) is sufficient to calculate the normalization factor, as the geNorm V value obtained for the 2 to 3 best genes (V_2-3_ = 0.0575) is lower than 0.15 and inclusion of additional genes would not significantly decrease the variation of the normalization factor (Figure 2).

Normfinder estimates inter- and intra-group variations and assigns a stability value for each gene based on a linear equation of the algorithm (see Methods). The results of Normfinder analysis for stable gene selection were different from those of geNorm. According to Normfinder, *Hprt1* was shown to be the most stable gene (0.049), and *Hprt1* and *Rpl5* represented the best combination of two genes (0.042) (Figure 1).

### Quantification of genes of interest: *Bnp, Anp, Mcp-1 and Nox-2* mRNA Expression in LVs

**Figure 3:**
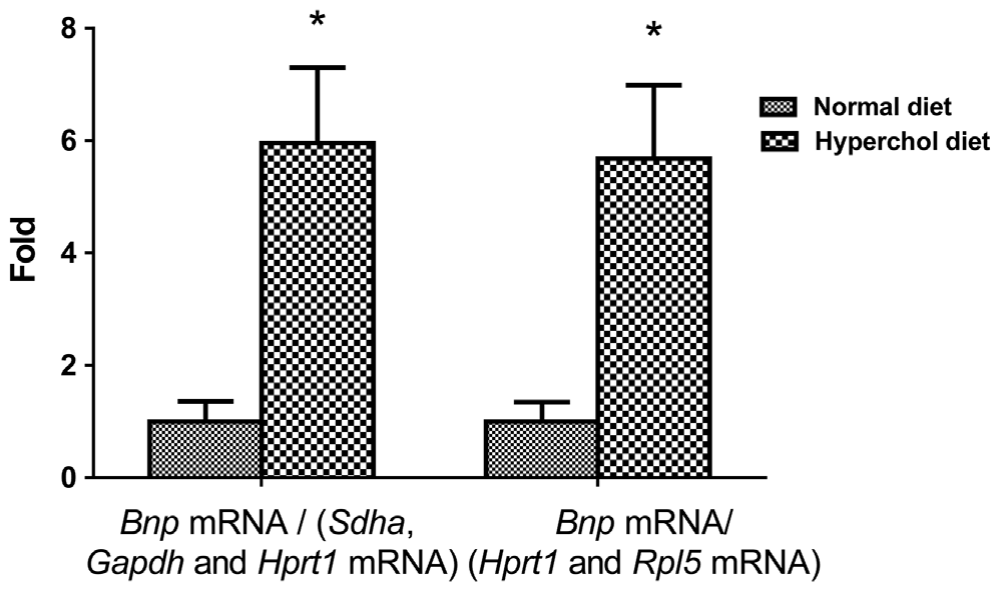
*Bnp* mRNA levels in the normal (n = 7) and high-cholesterol diet (n = 11) groups were normalised against geNorm (on the left) and Normfinder (right)-selected genes. Results shown correspond to mean ± SEM. Non-parametric Mann-Whitney test was used to compare the groups (*P<0.02).

The mRNA expression of the genes of interest was corrected using the reference genes selected from both normalization software (geNorm and Normfinder). Using both combinations suggested by the two software for normalization, we confirm that *Bnp* mRNA expression was induced in LVs from the hypercholesterolemic group compared to the normal group (Figure 3). Indeed, when we normalized against the geometric mean of *Sdha*, *Gapdh and Hprt1* (selected by geNorm), *Bnp* expression was found to be 5.96 fold increased in the hypercholesterolemic group compared to normals (*P* = 0.012). In agreement with this finding, the use of the geometric mean of *Hprt1* and *Rpl5* (selected by Normfinder) showed a 5.68 fold increase in *Bnp* expression in the hypercholesterolemic group (*P* = 0.019). When using the last gene from geNorm classification (*G6pd*), the *Bnp* increase was underestimated (4.2 fold, *P* = 0.011), whereas when normalizing against the last classified gene selected by Normfinder (*Eef1e1*), the *Bnp* increase was overestimated (6.84 fold, *P* = 0.011).

Also *Anp*, *Mcp-1* and *Nox-2* mRNA expressions were found to be increased in the hypercholesterolemic group compared to the normal group (Figure S2 in File S1). Similar results were obtained using both normalization strategies. However, when using *G6pd* for normalization, *Anp* and *Nox-2* expression increases were not significant between groups (*P* = 0.109 and *P* = 0.103, respectively).

**Figure 4:**
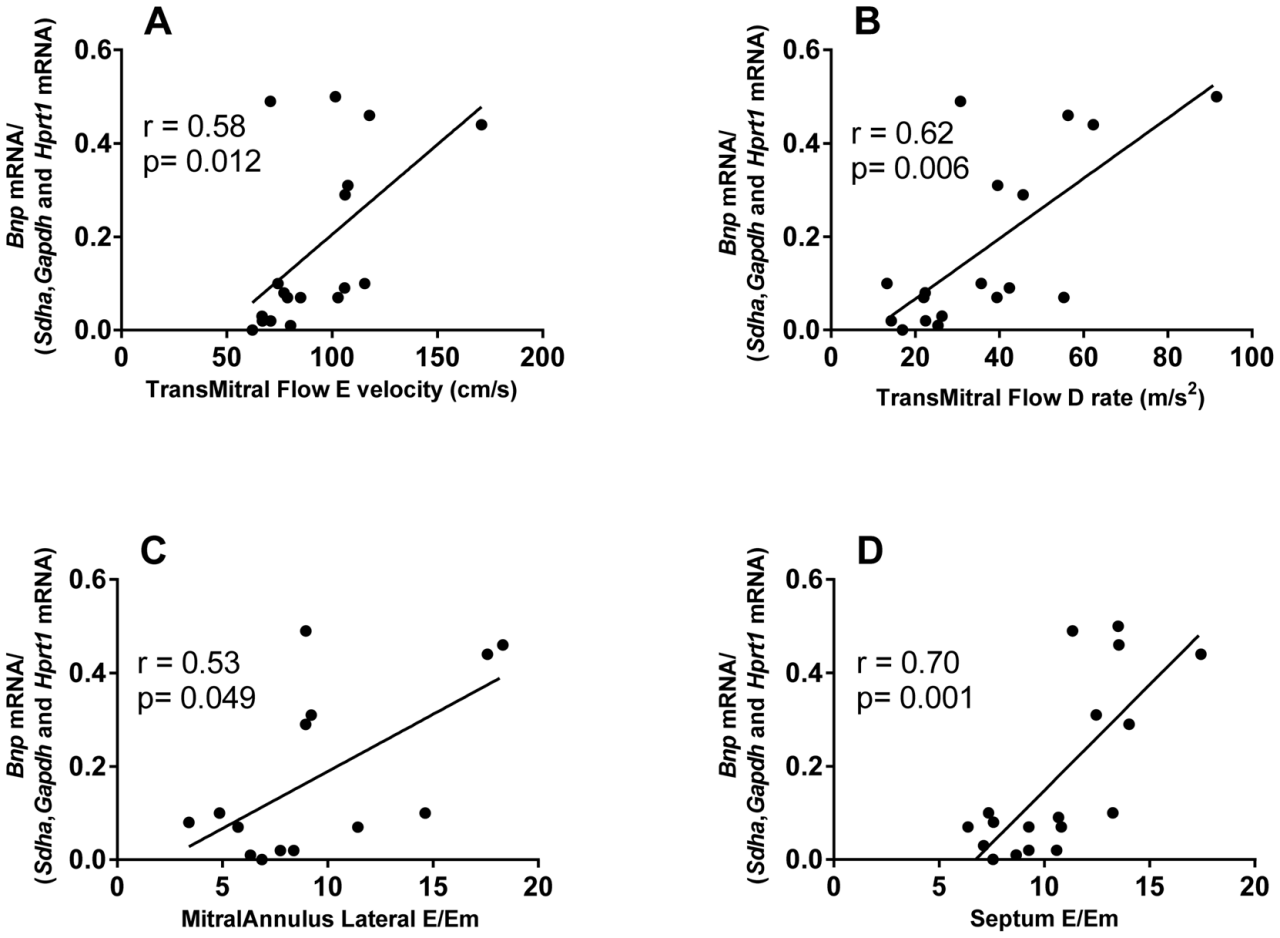
Spearman correlation between normalised *Bnp* mRNA levels (using geNorm selected reference genes) in rabbit left ventricles (normal diet n = 7 and cholesterol diet n = 11) and LVDD parameters determined by echocardiography before sacrifice: transmitral flow E velocity (A), transmitral flow deceleration rate (B), mitral annulus lateral E/Em ratio (C) and mitral annulus septal E/Em ratio (D).

Finally, we found that the mRNA expression of *Bnp* correlates with many LVDD-related echocardiographic parameters: transmitral flow E wave velocity, transmitral flow deceleration time, transmitral flow deceleration rate, transmitral flow A duration, left pulmonary vein (A-Ar) duration, right pulmonary vein (A-Ar) duration, mitral annulus lateral E/Em and septal E/Em ratio. Indeed, higher *Bnp* mRNA expression values correspond to higher LVDD severity. Using geNorm or Normfinder reference genes for normalization resulted in very similar correlations of *Bnp* with echocardiographic parameters. In Figure 4, we show some of these correlations using geometric mean of *Sdha*, *Gapdh* and *Hprt1* as examples.

## Discussion

### Echocardiographic Validation of a Rabbit Model of LVDD

Multiple echocardiographic parameters (including E-wave velocity, E-wave deceleration time, E-wave deceleration rate, difference in A-Ar durations and E/Em ratios) provide a robust demonstration of diet-induced restrictive LVDD in our rabbit model. Collectively, the changes reflect increased filling pressures, a stiffer LV and severe LVDD [27]. We believe that the proper selection of reference genes in this rabbit model will help further studies to investigate the different mechanisms that may be related to LVDD.

### RT-PCR Validation of a Rabbit Model of LVDD

Although hypercholesterolemia has been shown to promote LVDD [28], the mechanisms underlying this progression remain largely unknown. Indeed, some studies reported cellular mechanisms that may be altered in LVDD, including pathways involved in calcium homeostasis as well as extracellular matrix components [29]. Analysis of gene expression is essential to understand the pathways involved in LVDD. RT-PCR is used to validate and determine gene expression because of its high specificity, sensitivity and rapidity. Also, small amounts of samples are needed in such experiments, which facilitate the feasibility of these studies. RT-PCR normalization analysis is crucial for proper interpretation of gene expression data [18]–[21]. Our study provides the best combination of reference genes (*Sdha*, *Gapdh* and *Hprt1* by geNorm; *Hprt1* and *Rpl5* by Normfinder) that may be used in further mechanistic studies in our animal model. Indeed, these genes are involved in different pathways such as glycolysis, citric acid cycle, purine nucleotide synthesis and ribosomal RNA maturation and have been frequently used as reference genes in earlier studies. Among these studies, we particularly noticed those related to cardiomyocytes and heart failure in general [30]–[33]. It is important to note that due to the partially completed rabbit genome sequencing, assembly and annotation, finding reference sequences in the rabbit’s genome that correspond to reference genes is still difficult compared to humans, mice or rats. For this reason, we validated by sequencing the PCR products for each gene tested here. To our knowledge, this is the first report that gives validated reference genes in a rabbit model of LVDD but our results could also be of use for mRNA assessments from similar hypercholesterolemic rabbit models.

Considering the differences between the two methods used for reference gene selection, it was not surprising to obtain different stability rankings. Indeed, the geNorm algorithm is based on a pairwise variation test, which gives an overall stability of a gene in comparison to the rest of tested genes. However, the Normfinder algorithm estimates inter and intra-group variation and gives a final stability value based on these two parameters. Anderson and colleagues have demonstrated that Normfinder ranking is not similar to geNorm because of the differences between approaches [20]. However, other studies have showed similar classification by using the different approaches [34]. Vandesompele and colleagues concluded that each group has to validate their reference genes regardless of the method used; however, the method used should be reported [19]. When we used the best combinations of reference genes suggested by both methods, we obtained the same results regarding the increase of *Bnp* gene expression. However when using single non-validated potential reference genes (*G6pd* or *Eef1e1*), we showed that the fold increase results obtained were differing by 63%. Similar results and conclusions were obtained with *Anp*, *Mcp-1* and *Nox-2*. The use of a combination of reference genes resulting in stable normalization factors is crucial when changes between groups are not as high as what we observed in the case of *Bnp* mRNA expression. It is frequent in this type of studies to get modest differences between groups for many genes (≈ 2 folds) like *Nox-2* in our present study. In such cases, the validation of the reference genes is even more important.

BNP is a well-documented marker of the evolution of LV dysfunction, with the levels of BNP directly correlating with the severity of LVDD [12], [13]. Our findings further confirm this concept with the expression of *Bnp* (and the similar marker of disease *Anp* [10]) mRNA correlating with the echocardiographic results, thereby further validating our model of LVDD in hypercholesterolemic rabbits. Also, the increase of *Mcp-1* and *Nox-2* mRNAs validate the involvement of inflammation and oxidative stress in our model. These additional genes add evidence for the validity of our model as they have been reported to be differentially expressed in LVDD cases [9], [10]. Indeed, macrophage recruitment which is mediated in part by *Mcp-1* and adhesion molecules such as intracellular adhesion molecule 1 (ICAM-1) and vascular cell adhesion molecule 1 (VCAM-1), exerts a crucial role in myocardial fibrosis and diastolic dysfunction. Myocardial fibrosis and LVDD were improved by using an *Mcp-*1 neutralizing antibody [35]. Reactive oxygen species (ROS) derived from NADPH subunits such as Nox-2 contribute by several processes to the development of cardiac contractile dysfunction and remodelling [36]. *Gapdh* is often solely used to normalize RT-PCR data in many studies. However, it was clearly shown that *Gapdh* expression is modulated under certain conditions, especially in hypoxia [37], [38]. Surprisingly, normalizing only with *Gapdh* in our study yielded the same results in terms of *Bnp* mRNA expression modulation (5.71 fold increase, data not shown) as when using the geometric mean of our best genes combinations. This is probably due to the high stability of this gene in our model (geNorm stability: M<0.2) and because of the large difference in *Bnp* expression seen between the hypercholesterolemic and normal groups. Moreover, it is possible that hypoxia does not play a major role in this LVDD model.

## Conclusion

In the present study, we validated a hypercholesterolemic diet rabbit model of LVDD by echocardiography and by RT-PCR. We have identified the best reference genes (for normalization of RT-PCR data) that may be used in further studies to investigate mechanisms underlying LVDD.

## Supporting Information

File S1 **Figures S1 and S2.** Figure S1. Left ventricular diastolic dysfunction parameters assessed by echocardiography in both normal and hypercholesterolemic groups (A) Transmitral flow deceleration time (n = 7 and 11 for normal and hyperchol groups, respectively). (B) E/Em ratio (n = 7 for normal group and n = 10, 8, 7 for hyperchol group at time 0, 14.5 and 16.5 weeks, respectively). The first time-point corresponds to the beginning of the cholesterol-enriched diet for the hyperchol diet rabbit group (baseline); the rabbits from this group were given cholesterol-enriched diet for an average of 14.5 weeks, the time it took to develop significant aortic valve stenosis as defined by higher than 10% decrease in aortic valve area assessed by echocardiography, after which the hyperchol group of rabbits was switched to a normal diet for two weeks before sacrifice. *P<0.05; **P<0.01; ***P<0.001. E: peak velocity during early left ventricular filling, Em: mitral annulus velocity during early left ventricular filling. Figure S2. mRNA levels of *Anp* (A), *Mcp-1* (B) *and Nox-2* (C) in the normal (n = 7) and high-cholesterol diet (n = 11 except for *Anp* where n = 10) groups normalised against geNorm (on the left of each panel) and Normfinder (right)-selected genes. Results shown correspond to mean ± SEM. Non-parametric Mann-Whitney test was used to compare the groups. *P<0.05, **P<0.01, ***P<0.001.
(DOCX)

File S2 (DOCX)
