# Supplementary material for: Optimisation of Reference Genes for Gene-Expression Analysis in a Rabbit Model of Left Ventricular Diastolic Dysfunction
Source: PLoS One. 2014 Feb 18;9(2):e89331. doi: 10.1371/journal.pone.0089331 (PMC3928441; doi:10.1371/journal.pone.0089331)
Supplement: File S1 — Figures S1 and S2. Figure S1. Left ventricular diastolic dysfunction parameters assessed by echocardiography in both normal and hypercholesterolemic groups (A) Transmitral flow deceleration time (n = 7 and 11 for normal and hyperchol groups, respectively). (B) E/Em ratio (n = 7 for normal group and n = 10, 8, 7 for hyperchol group at time 0, 14.5 and 16.5 weeks, respectively). The first time-point corresponds to the beginning of the cholesterol-enriched diet for the hyperchol diet rabbit group (baseline); the rabbits from this group were given cholesterol-enriched diet for an average of 14.5 weeks, the time it took to develop significant aortic valve stenosis as defined by higher than 10% decrease in aortic valve area assessed by echocardiography, after which the hyperchol group of rabbits was switched to a normal diet for two weeks before sacrifice. *P<0.05; **P<0.01; ***P<0.001. E: peak velocity during early left ventricular filling, Em: mitral annulus velocity during early left ventricular filling. Figure S2. mRNA levels of Anp (A), Mcp-1 (B) and Nox-2 (C) in the normal (n = 7) and high-cholesterol diet (n = 11 except for Anp where n = 10) groups normalised against geNorm (on the left of each panel) and Normfinder (right)-selected genes. Results shown correspond to mean ± SEM. Non-parametric Mann-Whitney test was used to compare the groups. *P<0.05, **P<0.01, ***P<0.001. (DOCX) [file pone.0089331.s001.docx]

Supplementary File S1

Figure S1

Figure S2
