# Supplementary material for: Optimisation of Reference Genes for Gene-Expression Analysis in a Rabbit Model of Left Ventricular Diastolic Dysfunction
Source: PLoS One. 2014 Feb 18;9(2):e89331. doi: 10.1371/journal.pone.0089331 (PMC3928441; doi:10.1371/journal.pone.0089331)
Supplement: File S2 — (DOCX) [file pone.0089331.s002.docx]

**Supplementary File S2**

**Definitions:**

**Transmitral flow E wave velocity**: Measured by pulsed-wave Doppler, it indicates the maximum transmitral pressure gradient during early filling of the left ventricle (LV). This parameter is commonly used to classify the diastolic dysfunction (DD) stage. Early left ventricular filling remains active until the left ventricular pressure reaches that of the left atrium. The increase of E wave velocity means that the left atrial pressure is higher at the time of mitral valve opening, typically because the LV is stiffer. In patients with severe or restrictive stages of LVDD, this echocardiographic parameter is typically increased.

**Deceleration time**: The deceleration time of the transmitral flow E wave is the time taken from the maximum E velocity down to baseline (velocity of 0 cm/s). In severe LVDD, deceleration time is shorter because LV pressure reaches more rapidly left atrial pressure because LV compliance is reduced (LV is stiffer).

**E/Em ratio:** also called e/e’ or E/Ea ratio. Em is the early relaxation velocity measured by tissue Doppler. It can be measured at the mitral annulus lateral or septal region. The ratio of early transmitral flow velocity (E) to early diastolic mitral annulus velocity (E/Em) was shown to be the most accurate non-invasive predictor of elevated LV filling pressure, cardiac events and LVDD stage [1]. An increase of this ratio is seen in severe LVDD.

**Pulmonary vein Ar-Dur**: During left atrial contraction, a stiffer LV results in more marked reversal of flow going from the left atrium to the pulmonary veins. The duration of this reversed flow is measured by pulsed Doppler and called Ar-Dur. Higher Ar-Dur refers to larger reversal of flow and reflects a stiffer LV. This parameter was shown to be increased in severe stages of LVDD.**REFERENCE**

## 1. Kasner M, Westermann D, Steendijk P, Gaub R, Wilkenshoff U, et al. (2007) Utility of Doppler echocardiography and tissue Doppler imaging in the estimation of diastolic function in heart failure with normal ejection fraction: a comparative Doppler-conductance catheterization study. Circulation 116: 637-647.
